# The Position of Aβ_22−40_ and Aβ_1−42_ in Anionic Lipid Membranes Containing Cholesterol

**DOI:** 10.3390/membranes5040824

**Published:** 2015-11-30

**Authors:** Matthew A. Barrett, Richard J. Alsop, Thomas Hauß, Maikel C. Rheinstädter

**Affiliations:** 1 Soft Matter and Functional Materials, Helmholtz-Zentrum Berlin für Materialien und Energie, Lise-Meitner-Campus, Berlin, Germany; E-Mails: matthew.barrett@helmholtz-berlin.de (M.A.B.); hauss@helmholtz-berlin.de (T.H.); 2 Department of Physics and Astronomy, McMaster University, Hamilton, ON L8S 4M1, Canada; E-Mail: alsoprj@mcmaster.ca (R.J.A.)

**Keywords:** lipid membranes, cholesterol, Alzheimer’s disease, amyloid-β, X-ray diffraction

## Abstract

Amyloid-β peptides interact with cell membranes in the human brain and are associated with neurodegenerative diseases, such as Alzheimer’s disease. An emerging explanation of the molecular mechanism, which results in neurodegeneration, places the cause of neurotoxicity of the amyloid-β peptides on their potentially negative interaction with neuronal membranes. It is known that amyloid-β peptides interact with the membrane, modifying the membrane’s structural and dynamic properties. We present a series of X-ray diffraction experiments on anionic model lipid membranes containing various amounts of cholesterol. These experiments provide experimental evidence for an interaction of both the full length amyloid-$\beta_{1-42}$ peptide, and the peptide fragment amyloid-$\beta_{22-40}$ with anionic bilayer containing cholesterol. The location of the amyloid-β peptides was determined from these experiments, with the full length peptide embedding into the membrane, and the peptide fragment occupying 2 positions—on the membrane surface and embedded into the membrane core.

## 1. Introduction

Alzheimer’s disease is a degenerative brain illness, characterized by progressive deterioration of the brain. Although the disease has been studied for many years, the mechanism of neurotoxicity is still unclear. The first explanations placed the origin of the symptoms on “senile plaques”, proteinous deposits in the extracellular regions of the brain containing fibrillar amyloid-β (Aβ) peptides [1].

Although this hypothesis has been pursued, in recent years, a new hypothesis has emerged, placing toxicity on membrane active monomers and oligomers of Aβ [2,3,4,5,6]. Changes in the interactions between membrane lipids and proteins may be related to disease, with interactions leading to conformational changes and bilayer deformation. The amyloid-β peptide is cleaved in the membrane from the transmembrane protein amyloid precursor protein (APP), by β and γ secretases [7,8]. If the peptide remains or re-enters the membrane after cleavage, it has the potential to disrupt the structure and function of the membrane.

There is strong evidence that fragments of this peptide interact with lipid membranes, altering membrane structure [9,10], dynamics [11,12] and membrane function [13], thereby leading to changes in membrane permeability [14] and pore formation [15], although the exact mechanism remains obscure [16].

The synaptic plasma membrane of neuronal cells consists of a combination of charged and zwitterionic phospholipids, cholesterol and sphyingomylein. The link between cholesterol and Alzheimer’s disease was first introduced by Sparks *et al.* [17] but remains controversial [18,19,20]. Synaptic membranes are particularly rich in cholesterol, making up ∼25 mol % of membrane lipids [21]. Cholesterol helps to regulate the fluidity of the membrane and has implications in neural plasticity. Changes in the cholesterol concentration and distribution in cells has been linked to aging [22,23,24]. In aging cells, the amount of cholesterol increases, and cholesterol is redistributed between the inner and outer leaflets of the membrane [25]

High cholesterol is often considered a risk-factor in Alzheimer’s disease [26,27]. Patients with coronary artery disease (dense cholesterol plaques built up in the artery) have amyloid-β distribution in neuronal cells similar to those of AD patients [17]. A common treatment of high cholesterol, taking statins, has been linked to a diminished prevalence of Alzheimer’s disease. Cholesterol has been shown to promote the activity of β-secretase, increasing the production of amyloid-β from the amyloid precursor protein [28]. On the other hand, other studies have shown that neuronal cells of AD patients show cholesterol depletion [29], and it is thought that cholesterol protects against Aβ neurotoxicity.

While Aβ peptides are frequently reported in an extracellular location, A$\beta_{1-40}$ and A$\beta_{1-42}$ molecules were found to strongly interact with negatively charged lipids and to bind to anionic, negatively charged membranes [30,31,32,33,34,35,36,37], orienting parallel to the membrane surface. Studies on fragments of the amyloid-β peptide have shown that the peptide interacts with negatively charged membranes, entering the membrane and modifying membrane lipid dynamics [11,12]. The short, primarily hydrophobic (25–35) amino acid fragment embeds itself into the hydrophobic core of the membrane, and longer (1–40) or (1–42) peptides react in a similar way [38,39,40,41,42]. However, the interaction of the (22–40) fragment of the peptide, a fragment of similar length and hydrophobicity to the dimyristoylphosphatidylcholine (DMPC) lipid in membranes with high cholesterol content remains largely unknown. Additionally, the action of the full length peptide on membranes with various cholesterol content is not well understood.

We have performed a series of X-ray diffraction experiments in 1,2-dimyristol-sn-glycero- 3-phosphatidylcholine (DMPC) and 1,2-dimyristoyl-*sn*-glycero- 3-phospho-L-serine (DMPS) membranes to observe the equilibrium structure of an anionic lipid bilayer containing cholesterol and amyloid-β peptides. First, the cholesterol saturation limit of the anionic 92: 8 mol % DMPC/DMPS lipid bilayer was determined. Then, membranes containing a high, physiologically relevant, amount of cholesterol with amyloid-β peptides were created. This allowed us to study the interaction of the full-length (1–42) and the (22–40) fragment amyloid-β peptide in cholesterol rich anionic membranes.

## 2. Experimental Section

### 2.1. Membrane Samples Preparation

A series of lipid membrane samples were prepared for these experiments, with composition described in Table 1. The primary set of samples was used to determine the solubility ratio of cholesterol in a mixed (92 mol % DMPC: 8 mol % DMPS). A range of samples were produced with varying cholesterol content, from 0 mol % to 50 mol %. The lipids and cholesterol for each sample were deposited onto a silicon wafer to create a supported lipid multilayer of ∼3000 layers. A secondary set of samples was also produced, with a small amount of 1.5 mol % of amyloid-$\beta_{22-40}$ peptide with various cholesterol content. The samples were hydrated to 91% relative humidity in a sealed aluminum container with thin Kapton windows, and held at a constant temperature of 32 ${}^{\circ }$C during the measurements.

1,2-dimyristoyl -*sn*-glycero -3-phosphocholine (DMPC) and 1,2-dimyristoyl -*sn*-glycero -3-phospho -l-serine (DMPS) were purchased from Avanti Polar Lipids in lyophilized state and used as received. The full length amyloid-$\beta_{1-42}$ peptide (residues Asp-Ala-Glu-Phe-Arg-His-Asp-Ser-Gly-Tyr-Glu-Val-His-His-Gln-Lys-Leu-Val-Phe-Phe-Ala-Glu-Asp-Val-Gly-Ser-Asn-Lys-Gly-Ala-Ile-Ile-Gly-Leu-Met-Val-Gly-Gly-Val-Val-Ile-Ala) and also the fragment amyloid-$\beta_{22-40}$ (residues H- Glu- Asp- Val- Gly- Ser- Asn- Lys- Gly- Ala- Ile- Ile- Gly- Leu- Met- Val- Gly- Gly- Val- Val- OH) were purchased from Innovagen, Sweden (purity >95%) also in lyophilized form.

The zwitterionic lipid DMPC undergoes a main phase transition at ∼23 ${}^{\circ }C$, and the anionic lipid DMPS at 35 ${}^{\circ }C$ [43]. This DMPC/DMPS mixture is well suited for biophysical experiments because the phase transition temperatures fall into a range easily realizable in the laboratory. Our group has previously shown that a DMPC/DMPS (92: 8 mol %) lipid mixture undergoes a phase transition at ∼29 ${}^{\circ }C$, 98% relative humidity and the addition of 3 mol % A$\beta_{25-35}$ shifts this phase transition up to ∼32 ${}^{\circ }C$ [11]. In previous measurements, a peptide concentration of only ∼3 mol % has been shown to change membrane dynamics and also influence the phase transitions of the samples [11]. The addition of cholesterol to the system will result in the formation of a liquid-ordered (L${}_{o}$) phase.

**Table 1:** Composition of samples and measured membrane parameters. *d*: lamellar repeat distance calculated from the Bragg peaks found in the reflectivity , $d_{MM}$: maximum-to-maximum electron density distance as determined from a Fourier reconstruction, d${}_{water}$: water layer spacing; calculated from $d-d_{MM}$ and $A_{L}$: area per lipid, calculated from the in-plane Bragg rod location. (* with an uncertainty of ±0.1 Å)

DMPC	DMPS	Chol	A$\beta_{22-40}$	A$\beta_{1-42}$	*d* *	$d_{\mathrm{MM}}$ *	$d_{\mathrm{water}}$ *	$A_{L}$
(mol %)	(mol %)	(mol %)	(mol %)	(mol %)	(Å)	(Å)	(Å)	(Å${}^{2}$)
**Without peptide**								
92.0	8.0	0	-	-	50.3	35.2	15.1	46.0 ± 0.2
82.8	7.2	10	-	-	52.7	-	-	46.4 ± 0.4
64.4	5.6	30	-	-	54.3	39.7	14.6	46.7 ± 0. 1
59.8	5.2	35	-	-	54.3	-	-	47.3 ± 0.2
55.2	4.8	40	-	-	51.8	40.5	11.3	51.8 ± 0.4
46.0	4.0	50	-	-	51.4	-	-	53.6 ± 0.3
**A**$\beta_{22-40}$								
90.6	7.9	0	1.5	-	52.0	37.8	14.2	46.5 ± 0.1
63.4	5.5	29.6	1.5	-	53.2	40.0	13.2	50.2 ± 0.3
54.4	4.7	39.4	1.5	-	54.8	41.4	13.4	47.6 ± 0.1
45.3	3.9	49.3	1.5	-	58.4	-	-	50.5 ± 0.3
**A**$\beta_{1-42}$								
63.4	5.5	29.6	-	1.5	55.3	39.1	16.2	46.7 ± 0.2
54.4	4.7	39.4	-	1.5	54.6	40.4	14.2	47.6 ± 0.1
45.3	3.9	49.3	-	1.5	52.7	39.1	13.6	50.3 ± 0.3

The composition of each sample is listed in Table 1. The lipids were dissolved into solvent solution (80% chloroform, 20% methanol by volume), into a 15 mg/mL concentration lipids + cholesterol + peptide to solvent. For the samples containing amyloid-β, the peptide was dissolved into 0.5 mL of trifluoroacetic acid (TFA) which renders the peptide monomeric and prevents pre-aggregation before interaction with the membrane [44]. The TFA was evaporated in an Argon atmosphere for ten minutes. Following the TFA evaporation in the Aβ sample, the lipid solution was added. The solutions were sonicated for 2 minutes in a bath sonicator and vortexed to ensure complete mixing. The solution was deposited slowly onto 10 mm × 10 mm × 0.3 mm silicon slides using an artist’s airbrush, resulting in bilayers oriented parallel to the supporting solid wafer.

Before the experiment, the samples were incubated in a humidity and temperature controlled environment ((91 ± 1)% R.H. [45], (32 ± 1) ${}^{\circ }$C), for at least 12 h. The samples were then transferred into the X-ray humidity chamber with the same conditions, and allowed to re-equilibrate for 1 h before starting a scan. Both humidity environments were controlled with saturated salt solutions of Potassium Nitrate.

### 2.2. X-Ray Scattering

**Figure 1:**
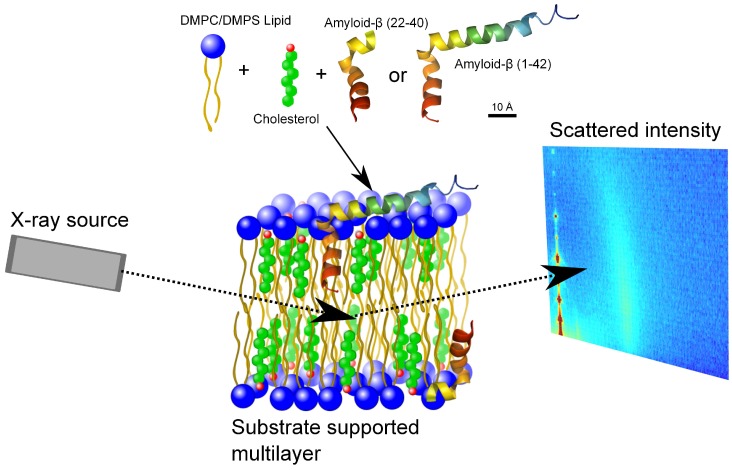
Schematic of the experimental setup. Membrane complexes were prepared with DMPC and DMPS lipids, cholesterol and amyloid-$\beta_{22-40}$ and amyloid-$\beta_{1-42}$ peptides. The samples were prepared as multiple lipid bilayers on a silicon substrate. The in- and out-of-plane X-ray scattering is gathered in a 2-dimensional reciprocal space map with a point detector.

X-ray diffraction data was obtained using the Biological Large Angle Diffraction Experiment (BLADE) in the Laboratory for Membrane and Protein Dynamics at McMaster University. BLADE uses a 9 kW (45 kV, 200 mA) CuK-*α* Rigaku Smartlab rotating anode at a wavelength of 1.5418 Å. Both source and detector are mounted on movable arms such that the membranes stay horizontal during the measurements. Focussing multi-layer optics provides a high intensity parallel beam with monochromatic X-ray intensities up to 10${}^{10}$ counts/(s × mm${}^{2}$). This beam geometry provides optimal illumination of the solid supported membrane samples to maximize the scattering signal. A sketch of the scattering geometry is shown in Figure 1. By using highly oriented membrane stacks, the in-plane ($q_{\left|\right|}$) and out-of-plane ($q_{z}$) structure of the membranes can be determined separately but simultaneously.

The result of such an X-ray experiment is a 2-dimensional intensity map of a large area (0.03 Å${}^{-1}<q_{z}<$ 1.1 Å${}^{-1}$ and 0 Å${}^{-1}<q_{\left|\right|}<$ 3.1 Å${}^{-1}$) of the reciprocal space, taking ∼18 h to acquire. The corresponding real-space length scales are determined by $d=2\pi /\left|Q\right|$ and cover length scales from about 2.5 to 60 Å, incorporating typical molecular dimensions and distances. These 2-dimensional data are essential to detect and identify signals from bilayers and peptides and determine orientation of the molecules. All scans were carried at (32± 1)${}^{\circ }$C and (91 ± 1)% RH. The membrane samples were mounted in a temperature and humidity controlled chamber, a so-called humidity chamber, during the measurements. Reflectivity scans were taken before and after the 18 h acquisition time to verify the stability of each sample during the measurement.

The 2-dimensional diffraction pattern can be directly used to determine the phase of the membrane lipids. A highly-ordered, gel-phase membrane results in vertical Bragg diffraction-rods indicating the crystallographic parameters of the lipid tails. Alternatively, for a more disordered membrane in the fluid-phase, these rods are smeared out into a broad, Gaussian-like peak. In our samples, the rods are not to be seen, which indicates fluid-phase membrane. Alternative methods to determine the membrane lipid phase would include fluorescence anisotropy measurements on membrane vesicles or deuterium NMR on deuterated lipids.

## 3. Results and Discussion

The molecular structure of multi-lamellar lipid stacks on silicon wafers was investigated using X-ray diffraction. The membranes are well oriented in lamellar structures allowing for information in and out of the lipid membrane plane to be collected simultaneously. Data from each sample were collected with the sample hydrated to 91% relative humidity at a constant temperature of 32 ${}^{\circ }$C. This relates to the fluid phase of the pure lipid sample, and the liquid-ordered phase of the samples containing cholesterol.

**Figure 2:**
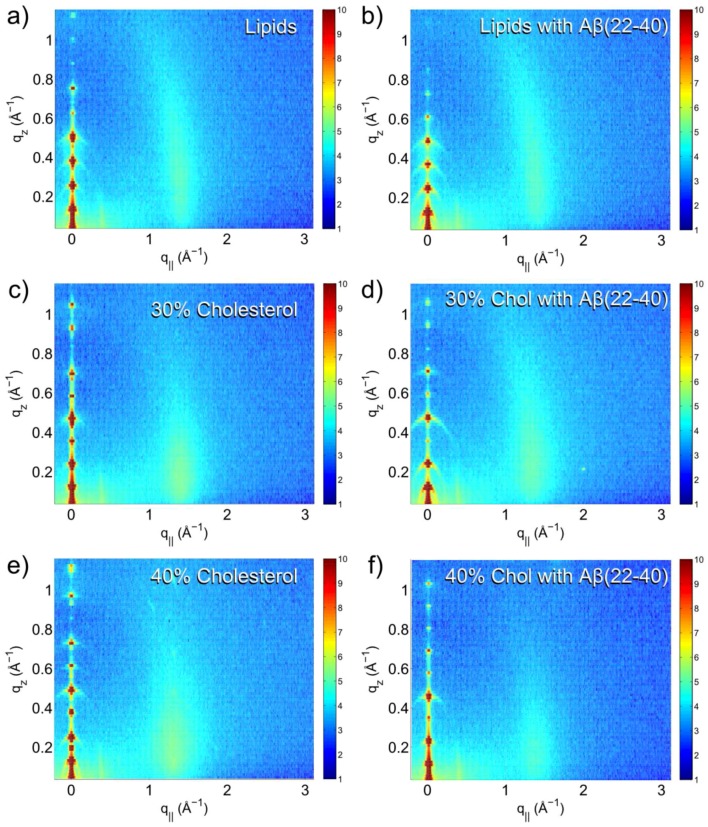
Reciprocal space maps of (**a**) DMPC/DMPS (92/8%) lipids; (**b**) lipid with 1.5 mol % amyloid-$\beta_{22-40}$; (**c**) lipids with 30 mol % cholesterol; (**d**) lipids with 30 mol % cholesterol and 1.5 mol % amyloid-$\beta_{22-40}$; (**e**) lipids with 40 mol % cholesterol and (**f**) lipids with 40 mol % cholesterol and 1.5 mol % amyloid-β (22–40).

Scans of each sample were obtained, covering an area of reciprocal space bounded by −0.3 Å${}^{-1}$ ≤ q${}_{\left|\right|}$ ≤ 3 Å${}^{-1}$ and 0 Å${}^{-1}$ ≤ q${}_{z}$ ≤ 1.1 Å${}^{-1}$. This type of scan results in a reciprocal space map, with features indicating structural details of the lipid bilayer. A selection of typical reciprocal space maps is shown in Figure 2.

In each of the reciprocal space maps collected, a series of well spaced Bragg peaks centered at q${}_{\left|\right|}$ = 0 Å${}^{-1}$ arise from the lamellar structure of the sample. These reflectivity peaks give information on the repeat distance of the bilayer, and of the out-of-plane structure of the bilayer through calculation of the electron density. Also visible in the reciprocal space map is a rod-like diffuse scattering intensity centered between 1 Å${}^{-1}$ and 2 Å${}^{-1}$ in q${}_{\left|\right|}$. Rod-like scattering is typical of a 2-dimensional system, and arises from the in-plane structure of the lipid bilayer, in particular the chain-correlation peak, which is the result of the packing of the lipid tails in the hydrophobic membrane core. In the well-ordered gel phase of the lipids, a sharp scattering is observed, taking the shape of a narrow peak profile. In this case, the chain-correlation peak is broad, which suggests that the samples are in the disordered fluid phase.

**Figure 3:**
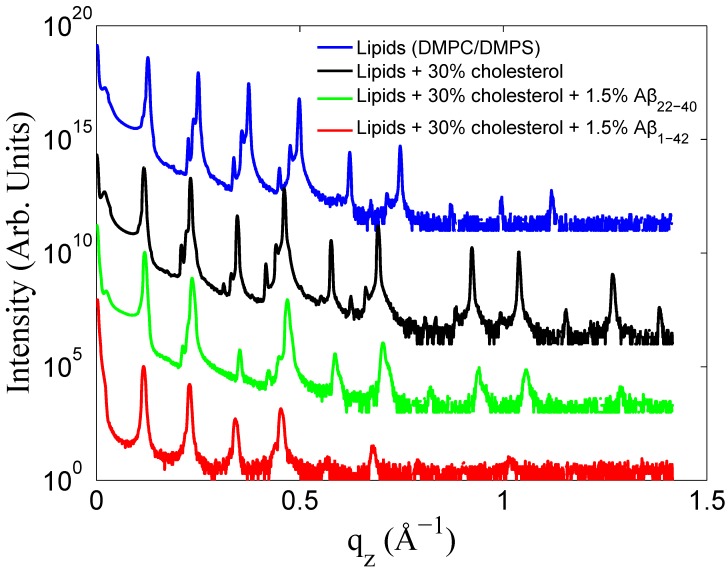
Reflectivity data from various data sets presented with logarithmic intensity. Lipids (92 mol % DMPC: 8 mol %DMPS) in blue, lipids with 30 mol % cholesterol in black, lipids with 30 mol % cholesterol and 1.5 mol % A$\beta_{22-40}$ in green, and lipids with 30% cholesterol and 1.5 mol % A$\beta_{1-42}$ in red. Curves have been rescaled in intensity for clarity.

**Figure 4:**
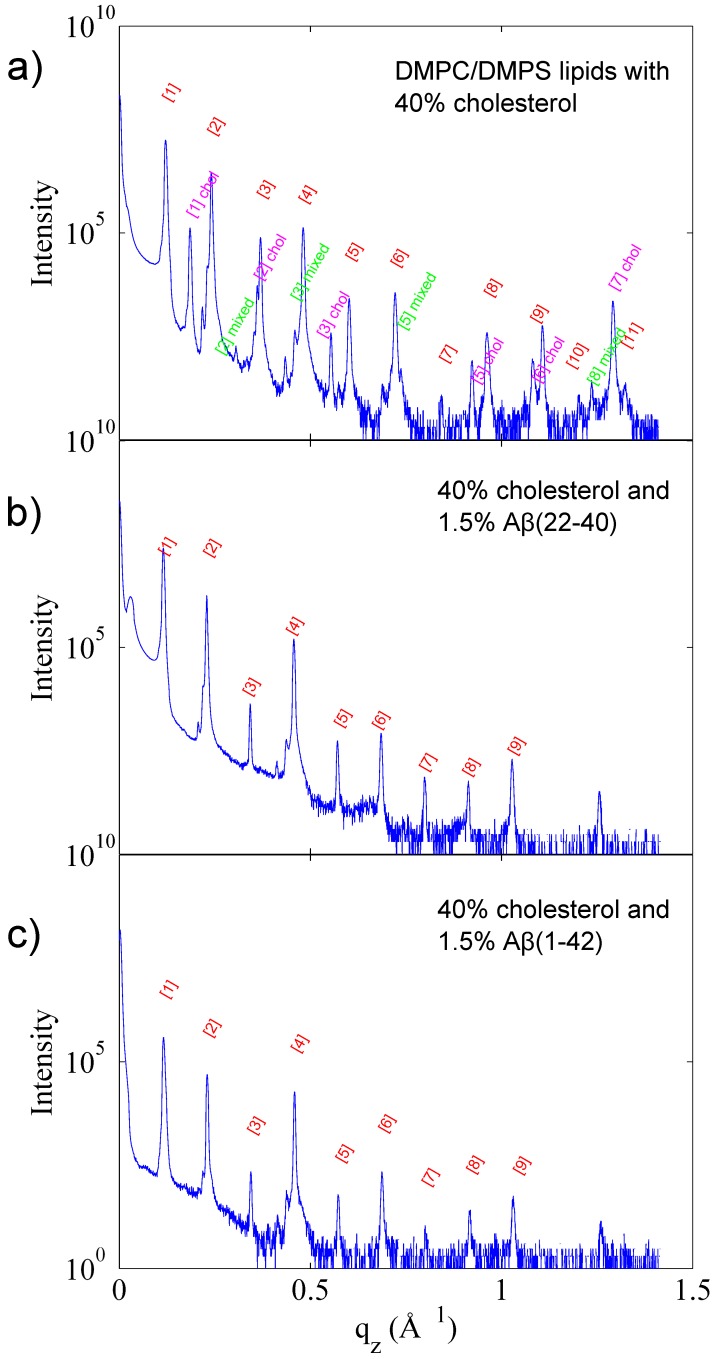
Reflectivity and peak assignment of (**a**) DMPC/DMPS with 40 mol % cholesterol; (**b**) DMPC/DMPS with 40 mol % cholesterol and 1.5 mol % A$\beta_{22-40}$ and (**c**) DMPC/DMPS with 40 mol % cholesterol and 1.5 mol % A$\beta_{1-42}$. Peaks which correspond to a lamellar phase are presented in red. In the sample without peptide (**a**), peaks relating to a full cholesterol bilayer (pink), and a mixed lipid and cholesterol bilayer (green) are also observed.

Selected reflectivity curves are presented in Figure 3. Up to 13 Bragg orders were observed indicative that all membrane complexes formed well developed lamellar structures. Lamellar peaks are indexed in Figure 4. The small side peaks are spurious reflections arising from the focussing optics and have been omitted from the data analysis. Additional peaks were observed in the 40 mol % cholesterol membranes. These peaks could be assigned to a pure cholesterol bilayer (with a *d*-spacing of 34 Å) indicative of a mixed phase, with the *d*-spacing corresponding the combination of 1/2 the cholesterol *d*-spacing and 1/2 the lipid *d*-spacing. This type of peak analysis has previously been observed with high cholesterol content DMPC bilayers [42,46].

**Figure 5:**
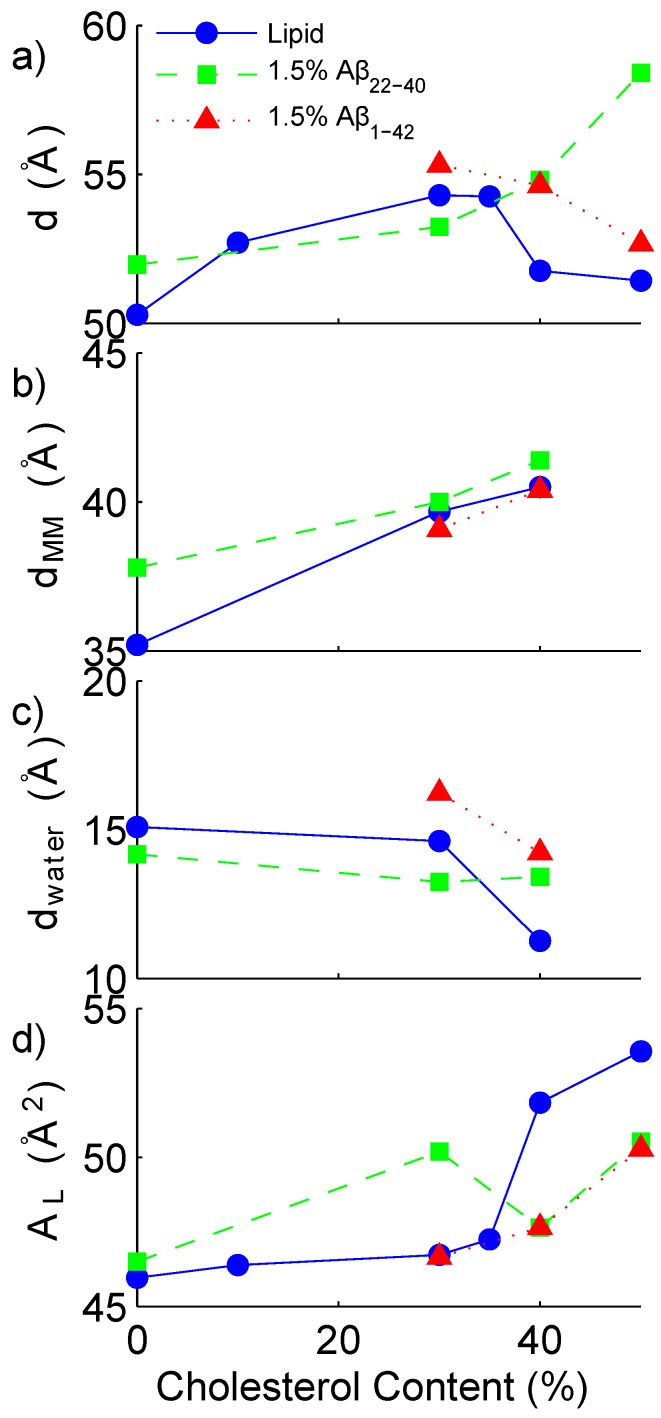
Cholesterol dependence of bilayer parameters. (**a**) lamellar repeat distance; (**b**) lipid head-to-head distance; (**c**) water layer thickness and (**d**) area per lipid. Lipid sample series (92 mol % DMPC: 8 mol % DMPC) represented with a solid line and blue symbols, samples containing 1.5 mol % A$\beta_{22-40}$ represented by a dashed line and green symbols and samples containing 1.5 mol % A$\beta_{1-42}$ represented by a dotted line and red symbols.

The out-of-plane electron density profile relating to each sample was reconstructed through Fourier transform. By integrating the peaks associated with the lamellar bilayer, the relative intensity of each Fourier contribution is determined. The reconstruction allows one to determine additional bilayer parameters, such as the water-layer spacing, and the head-to-head spacing of the bilayer. From the electron density profile, the highest peak (at *z*∼20 Å) is associated with the electron rich head group of the lipid. The maximum-to-maximum distance is calculated as twice the distance from the centre of the bilayer to this maximum. A simple approximation of the thickness of the water layer is then calculated by subtracting the maximum-maximum distance from the total *d*-spacing. In Figure 5, the total *d*-spacing, the maximum to maximum electron density distance (d${}_{MM}$) and the water layer thickness (d${}_{water}$) are presented as a function of cholesterol. d${}_{MM}$ can be thought of as an estimate of the head-to-head distance for a bilayer without peptides, however, may be affected by the peptide’s contribution to the electron density. The water layer thickness is then calculated as the total repeat distance minus d${}_{MM}$. Also presented in Figure 5 is the area per lipid molecule, (A${}_{L}$). This is calculated from the centre of the Gaussian fit of the rod-like intensity (q${}_{T}$) seen in the reciprocal space maps in Figure 2, that is, $A_{L}=16\pi^{2}/\left(\sqrt{3}{q_{T}}^{2}\right)$ [46,47,48]. All experimentally determined parameters are listed in Table 1. The values fall within a comparable range as previous measurements of DMPC in similar conditions [46,49].

By observing differences in the Fourier reconstruction of related samples, the structural details of the bilayer can be determined. The electron density, ρ(z), is approximated by a 1-dimensional Fourier analysis:

(1) $$\rho \left(z\right)=\frac{2}{d_{z}}\sum_{n=1}^{N}\sqrt{I_{n}q_{n}}\nu_{n}cos\left(q_{n}z\right)$$

*N* is the highest peak order detected and $d_{z}$ is the experimentally determined bilayer repeat distance. The integrated peak intensities, $I_{n}$, are multiplied by $q_{n}$, and the square-root of this value (the Lorentz factor) is multiplied by the phase coefficients, $\nu_{n}$, to yield the structure factor. A phase array of [-1 -1 1 -1 1 -1 1 -1 1 1 -1] was determined. For further details, refer to the Materials and Methods section of Reference [46] and references therein. To compare two electron density profiles, the profiles must be scaled appropriately. The bilayer is scaled by making an estimate of the electron density of a unit cell based on the number of electrons in the lipids, cholesterol, peptides and water molecules, which make up the unit cell and by determining the size of the unit cell represented by these molecules. The volume of the unit cell is calculated from the experimentally determined *d*-spacing, as well as the experimentally determined area per lipid. The area per lipid is smaller than that of a single component DMPC lipid sample [50]. The centre of the lipid bilayer should have contributions from the methyl group at the end of the lipid tails, with an electron density of 0.22 e${}^{-}$/Å${}^{3}$, only.

This scaling resulted in electron density profiles where the difference between a sample with and without peptide fluctuate around zero, due to the addition of the low concentration of peptide added. An additional scaling of the samples containing peptide was necessary to correct for two factors: the change in size of the unit cell (as determined from *d* and A*L*) and the lower lipid content of the sample. When scaled, the electron density profile of the samples with peptide have higher electron density than the samples without, and a subtraction can be made. This factor ranged from 1.07 for the amyloid-$\beta_{22-40}$ in 30 mol % cholesterol sample to 1.03 in the amyloid-$\beta_{1-42}$ in 40 mol % cholesterol sample. This scaling strategy was recently used by Alsop *et al.* [51].

**Figure 6:**
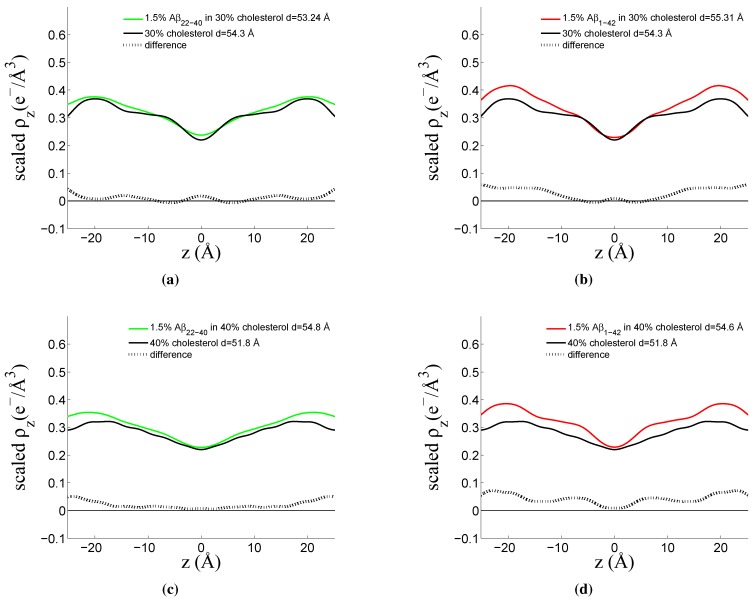
Fourier reconstructions of out-of-plane bilayer structure. (**a**) DMPC/DMPS membrane with 30 mol % cholesterol (black line) and 1.5 mol % amyloid-$\beta_{22-40}$ (green line) and difference (black dotted line); (**b**) DMPC/DMPS membrane with 30 mol % cholesterol (black line) and 1.5 mol % amyloid-$\beta_{1-42}$ (red line) and difference (black dotted line); (**c**) DMPC/DMPS membrane with 40 mol % cholesterol (black line) and 1.5 mol % amyloid-$\beta_{22-40}$ (green line) and difference (black dotted line); (**d**) DMPC/DMPS membrane with 40 mol % cholesterol (black line) and 1.5 mol % amyloid-$\beta_{1-42}$ (red line) and difference (black dotted line).

The rescaled electron densities of a 30 mol % and 40 mol % cholesterol sample containing both the peptide fragment amyloid-$\beta_{22-40}$, and the full-length peptide amyloid-$\beta_{1-42}$ are presented in Figure 6, respectively.

**Figure 7:**
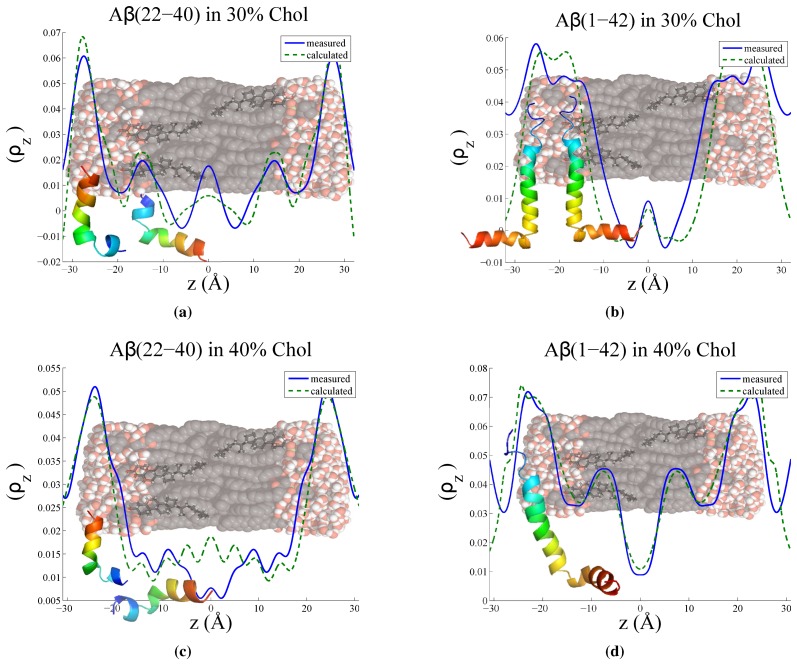
Measured electron density difference between sample with and without peptide (blue solid line) and model calculation (green dashed line) for (**a**) 30 mol % cholesterol with A$\beta_{22-40}$ peptide fragment; (**b**) 30 mol % cholesterol with A$\beta_{1-42}$ full-length peptide; (**c**) 40 mol % cholesterol with A$\beta_{22-40}$ peptide fragment; (**d**) 40 mol % cholesterol with A$\beta_{1-42}$ full-length peptide. Models were calculated by determing the electron density profile of solution protein structures from the protein data bank (reference 1IYT), and determining a reasonable orientation in the membrane which resembles the measured electron density.

To determine more accurately the position and orientation of the peptides in the lipid bilayer, the difference between the two curves was calculated and the resulting electron density difference compared to a curve calculated from a model. A protein structure for the full-length peptide was obtained from the Protein Data Bank (reference 1IYT [52]). The amyloid-$\beta_{22-40}$ peptide fragment structure was estimated by cutting the full-length peptide, as no solution structure has been determined for this particular fragment to our knowledge. The position of each atom’s electrons in the out-of-plane direction is projected onto the *z*-axis as a Gaussian distribution (accounting for thermal fluctuations) and through rotation and translation of the peptide, a model is formed and the electron profile is calculated. This approach is further explained in detail by Dies *et al.* [42]. Figure 7 shows the measured electron density difference and the electron profile calculated from the modeling.

We note that if the bilayer structure is significantly changed by the presence of the peptides, a direct subtraction may not give information about the peptide location or orientation. A more direct method to localize peptide location is using neutron diffraction in combination with site-specific deuterium labelling, in which a comparison between a membrane structure with unlabelled peptide and deuterium-labelled peptide is possible. With this approach, the position of the fragment amyloid-$\beta_{25-35}$ was determined in a model membrane composed of 1-palmitoyl-2-oleoyl-sn-glycero-3-phosphocholine (POPC) and 1-palmitoyl-2-oleoyl-sn-glycero-3-phospho-L-serine (POPS) [9]. In this work it was found that the membrane structure itself remains essentially unperturbed in the presence of the amyloid-$\beta_{25-35}$ peptide fragment. The same result has also been observed by our group for membranes in the presence of amyloid-$\beta_{22-40}$ (unpublished result). In these cases, the observed changes include a slight broadening of the lipid head-group region, and an isotropic smoothing of the neutron scattering length density of the lipid acyl-chain region.

However, neutron diffraction experiments are often limited by the low neutron flux and correspondingly small number of higher order reflections, which limits the resolution of the Fourier transform. Our X-ray experiments show up to 13 Bragg orders. The corresponding electron densities have an unprecedented spatial resolution such that we can compare the experimentally determined electron density with the calculated electron density of the peptide molecules. This novel approach enables us to determine the position and orientation of the peptides, which is complementary to the position of a label determined by neutron diffraction.

### 3.1. Solubility Limit of Cholesterol in 92 mol %DMPC:8 mol %DMPS Bilayer

Reflectivity curves indicate a lamellar lipid phase for all samples. The small peak intensities at lower q${}_{z}$ are attributed to spurious reflections from the focussing optics and are omitted in the data analysis. For samples with a cholesterol content greater than 40 mol %, additional peaks associated with cholesterol plaques resulting in a mixed lipid/cholesterol phase are also observed. This saturation of cholesterol in the lipid bilayer has been reported for a DMPC bilayer, and occurs at 37.5 mol % cholesterol [46]. It has also been observed in an anionic lipid bilayer at 30 mol % cholesterol by Dies *et al.* [42].

The samples consisting of only lipids and cholesterol (without peptide) exhibit a lamellar repeat distance (*d*-spacing) cholesterol dependence which increases slightly with increasing cholesterol content in the unsaturated (low ratio) cholesterol regime, as is expected for a fluid-phase saturated lipid bilayer [53]. This is cholesterol’s well known condensation effect. At 40 mol % cholesterol, a drop in *d*-spacing is observed, coincident with the appearance of the peaks relating to saturated cholesterol plaques. The 50 mol % cholesterol sample exhibits a similar *d*-spacing to the 40 mol % sample, which is explained by the excess cholesterol molecules joining the already present cholesterol plaques.

### 3.2. Increased Cholesterol Solubility Limit in the Presence of Amyloid-*β* Peptides

Samples containing 1.5 mol % of either the amyloid-$\beta_{22-40}$ peptide fragment or the amyloid-$\beta_{1-42}$ full length peptide did not exhibit the fingerprint of cholesterol plaques in the reflectivity profile for samples containing high cholesterol content.

For samples with the (22–40) fragment present, the *d*-spacing increases with cholesterol content above 40 mol %. The peptide seems to prevent the formation of insoluble cholesterol plaques, and the trend of cholesterol to increase *d*-spacing continues. Instead of reaching the solubility limit of cholesterol in the membrane at ∼40%, as in the peptide-free case, the presence of the peptide disturbs the regular packing of the lipid matrix, increasing the amount of cholesterol the lipids can host before saturation occurs. The trend of increasing bilayer thickness continues, and the *d*-spacing increases to 58.4 Å with 50 mol % cholesterol.

For the full-length amyloid-$\beta_{1-42}$ samples, the *d*-spacing decreases smoothly with increasing cholesterol content from 30–50 mol %. As shown in Figure 7, this effect may be a result of the hydrophobic portion of the peptide embedding into the bilayer with the longer end of the peptide residing on the surface of the bilayer. Similar to the amyloid-$\beta_{22-40}$ peptide result, the regular packing of the lipid matrix may be disrupted by the addition of the peptide, allowing the bilayer to host more cholesterol before saturation. The decrease in *d*-spacing with increased cholesterol content in the sample may be related to the effect of the full-length peptide promoting fusion between bilayers. Experimental evidence has been presented by Dante *et al.* [10]. A decrease in the water-thickness is seen between 30 mol % and 40 mol % cholesterol, which may hint at the thinning of the water layer by an electrostatic effect of the long surface-present peptide segment.

The lack of cholesterol peaks present in the reflectivity curves is in contrast to the findings of Dies *et al.* [42], showing that the presence of the amyloid-$\beta_{25-35}$ peptide fragment promotes insoluble cholesterol plaque formation; however, the length and the hydrophobicity profile of the (25–35) peptide fragment is quite similar to that of cholesterol. In contrast to the amyloid-$\beta_{22-40}$ and the amyloid-$\beta_{1-42}$ peptides in use in this study, the (25–35) peptide contains primarially hydrophobic peptides and may recruit cholesterol to form domains, whereas the two peptides studied in our paper contain also hydrophilic residues.

### 3.3. Position of Amyloid-*β* Peptide Fragments in the Bilayer

Through a Fourier reconstruction of the lamellar scattering out-of-plane of the membrane, a selection of samples were further analyzed. Samples containing the peptide fragment (residues 22–40) and the full-length peptide (residues 1–42) were compared with the lipid and cholesterol bilayer above and below the saturation limit of cholesterol in the anionic bilayer. The electron density profiles of each of the non-peptide samples were subtracted from the associated electron density profiles containing the peptide. A modeling procedure was carried out to interpret the complex electron density difference profile associated with the peptides.

The reconstruction of the cholesterol-free membrane containing amyloid-$\beta_{22-40}$ was performed, but the two peptide position closely resembles what is reported upon below for the membranes with cholesterol, and thus will not be discussed in greater detail. The position of the amyloid-$\beta_{1-42}$ peptide in a cholesterol-free membrane has been reported upon previously using X-ray diffraction [42]. The positions of the peptides in cholesterol rich membranes are reported below.

#### 3.3.1. Peptide Fragment: Amyloid-$\beta_{22-40}$

The model which best describes the location of the (22–40) residue peptide fragment in Figure 7a and c consists of a two position peptide distribution. In both the 30 mol % cholesterol and the 40 mol % cholesterol cases, one population of peptide sits parallel to the lipid tails, whereas the other population lies perpendicular to the bilayer normal, resting above the lipid head-groups. At 40 mol % cholesterol content, the peptide tilt-angle in the bilayer changes, adopting a position in which the longest end-to-end axis of the peptide is parallel with the tails. No significant change in the peptide on the head-groups is observed.

This finding is supported by the results of previous X-ray and neutron diffraction studies with deuterium labelled amyloid-$\beta_{25-35}$ [42,54]. In the X-ray profile, two surface bound states of the peptide were observed, with an additional embedded peptide state. In the neutron scattering study, at low cholesterol content, two peptide populations were observed, in similar locations. The model involves a ratio between the number surface and core peptide populations. At 30 mol % cholesterol, the model used was a ratio of 55:45 surface:embedded and at 40 mol % cholesterol, the surface bound peptide population increased, resulting in a ratio of 70:30 surface to embedded. This trend was also observed in the neutron scattering experiment, with high cholesterol content (20 mol % cholesterol) preventing further intercalation of the peptide into the bilayer. The difference between the amount of cholesterol may be related to the use of POPC, rather than DMPC as the main bilayer constituent.

A two peptide location model may also be related to the two stage model of peptide/membrane interaction [55,56,57]. The first stage of peptide interaction involves contact with the membrane, with the peptide aligning itself parallel to the membrane surface. The second stage involves the peptide embedding itself into the membrane’s core. The increase in cholesterol content may increase the free-energy of insertion [58], resulting in an increase of surface peptides.

#### 3.3.2. Full Length Peptide:Amyloid-$\beta_{1-42}$

The location of the (1–42) residue full-length peptide in Figure 7b and d was modeled with the hydrophilic part of the peptide (starting with residue 1) occupying a position on the surface of the bilayer, and the hydrophobic part of the peptide residing in the membrane core, parallel to the lipid tails. For 30 mol % cholesterol model, it was necessary to use two populations of similarly oriented peptides to model the electron density difference. This can be physically interpreted as a contribution of the adjacent bilayer’s peptide to the unit cell. The 40 mol % cholesterol was modeled with a single peptide. The peptide was slightly tilted with respect to the bilayer and does not embed as deeply into the bilayer core, a result consistent with the peptide fragment model discussed previously.

A thinning of the water layer is also observed between the 30 mol % and 40 mol % cholesterol samples. The increased cholesterol content in the bilayer acting to expel the peptide from the bilayer centre may increase the action of the fusogenic activity of the peptide, which has been observed experimentally with small-angle neutron scattering on cholesterol free lipid samples [10]. The external segment of the peptide acts via electrostatics to decrease the water layer thickness and bring adjacent bilayers closer together. There is also evidence that the full-length peptide induces pore-formation in the membrane [59]. The structural disruption, formation of pores and the act of membrane fusion may go hand in hand to give rise to the neurotoxicity associated with the disease.

## 4. Conclusions

Experimental evidence for the interaction of the full-length amyloid-$\beta_{1-42}$ peptide and the peptide fragment amyloid-$\beta_{22-40}$ has been presented. We provide evidence that both peptides increase the solubility limit of cholesterol into an anionic 92 mol % DMPC:8 mol % DMPS bilayer, preventing the formation of cholesterol plaques. Both peptides embed into a lipid bilayer containing 30 mol % and 40 mol % cholesterol; however, the higher cholesterol content prevents the peptides from embedding deeply. The full length peptide extends into the bilayer, embedding into the hydrophobic core, whereas two populations of the peptide fragment (22–40) exist, one lying parallel to the membrane surface, and another embedded into the membrane core.

These results increase the understanding of the complexities of membrane and peptide interactions, and provide further evidence of the interaction of the Alzheimer’s peptide amyloid-β with model membranes. A better understanding of the structural behavior of peptides and model membranes may shed light on the molecular origin of symptoms and may lead to future prevention or early detection strategies.
